# Hydrophobic cue-induced appressorium formation depends on MoSep1-mediated MoRgs7 phosphorylation and internalization in *Magnaporthe oryzae*

**DOI:** 10.1371/journal.pgen.1010748

**Published:** 2023-05-15

**Authors:** Jiayun Xu, Xinyu Liu, Wei Zhang, Wanzhen Feng, Muxing Liu, Leiyun Yang, Zhixiang Yang, Haifeng Zhang, Zhengguang Zhang, Ping Wang

**Affiliations:** 1 Department of Plant Pathology, College of Plant Protection, Nanjing Agricultural University, and Key Laboratory of Integrated Management of Crop Diseases and Pests, Ministry of Education, Nanjing, China; 2 The Key Laboratory of Plant Immunity, Nanjing Agricultural University, Nanjing, China; 3 Department of Microbiology, Immunology, and Parasitology, Louisiana State University Health Sciences Center, New Orleans, Louisiana, United States of America; CAAS IPP: Chinese Academy of Agricultural Sciences Institute of Plant Protection, CHINA

## Abstract

The rice blast fungus *Magnaporthe oryzae* forms specialized infectious structures called appressoria that breach host cells to initiate infection. Previous studies demonstrated that the regulator of G-protein signaling (RGS)-like protein MoRgs7 undergoes endocytosis upon fungal sensing of hydrophobic environmental cues to activate cAMP signaling required for appressorium formation. However, the mechanism by which MoRgs7 internalizes and its fate remains undetermined. We here show that MoSep1, a conserved protein kinase of Mitotic Exit Network (MEN), phosphorylates MoRgs7 to regulate its function. MoRgs7 phosphorylation determines its interaction with MoCrn1, a coronin-like actin-binding protein homolog that also modulates the internalization of MoRgs7. Importantly, the endocytic transport of MoRgs7 is critical for its GTPase-activating protein (GAP) function important in cAMP signaling. Together, our findings revealed a novel mechanism by which *M*. *oryzae* activates MoRgs7-mediated hydrophobic cue-sensing signal transduction involving protein phosphorylation and endocytic transport to govern appressorium formation and fungal pathogenicity.

## Author summary

During its pathogenic interaction with rice, *M*. *oryzae* forms a highly specialized infectious structure called appressorium to initiate infection. Previous studies showed that the seven transmembrane (7-TM) domain-containing regulators of G-protein signaling (RGS)-like protein MoRgs7 is vital for the perception of environmental cues and cAMP-dependent appressorium formation. However, little is known about how MoRgs7 transduces hydrophobic cues and their regulation. In this study, we have identified that MoSep1 phosphorylates MoRgs7 during appressorium formation, and phosphorylation of MoRgs7 leads to a high binding affinity with MoCrn1 and its internalization. Additionally, the internalization of MoRgs7 contributes to the activation of cAMP signaling, further governing appressorium formation and pathogenicity.

## Introduction

*Magnaporthe oryzae* poses the most severe threat to rice production across the world and is also an excellent model organism for studying plant–pathogen interactions [1, 2]. *M*. *oryzae* produces the appressorium, a unique infectious structure, following perceiving host-surface characteristics, and from which the invasive hyphae emerge to penetrate the host cells [3–5]. It is suggested that *M*. *oryzae* activates downstream effectors to trigger appressorium development by sensing and internalizing surface hydrophobicity and hardness cues [3, 6]. G-protein-coupled receptors (GPCRs) with seven transmembrane domains (7-TM) are the largest family of membrane receptors that sense extracellular surface cues to activate G proteins and downstream effectors [7]. The first identified GPCR protein in *M*. *oryzae* is MoAci1 that is involved in signal recognition and interaction with the adenylate cyclase MoMac1 [8]. MoPTH11, also a G-protein coupled receptor, senses the hydrophobic leaf surface during appressorium differentiation [9]. Additionally, MoSho1 is involved in the perception of host surface hydrophobicity and phosphorylation of the MAP kinase MoPmk1 [10]. MoSho1 is essential for appressorium formation and pathogenicity [11]. Moreover, MoWish, also a conserved seven-transmembrane receptor protein, internalizes hydrophobicity signals and modulates cAMP- and PMK1/MAPK signaling [12]. Despite the findings of the above-mentioned GPCRs in activating signaling pathways during appressorium formation, the process by which the receptor internalizes signaling from the plasma membrane (PM) is not known.

Internalization of GPCRs is fundamental to maintaining cell responsiveness and homeostasis through the spatial regulation of signals. Multiple scenarios of signaling internalization were discovered in several model organisms. In the budding yeast *Saccharomyces cerevisiae*, the pheromone receptor Ste2p protein is phosphorylated upon binding of the pheromone α-factor, and the C-terminal S/T phosphorylation sites are thought to be involved in the α-factor-induced internalization of Ste2p [13]. In *Arabidopsis thaliana*, AtRGS1, containing a predicted 7-TM domain and presumably a glucose receptor or co-receptor, is subject to regulation by AtWNK8, a member of WITH NO LYSINE (WNK) family Ser/Thr kinases. AtWNK8 phosphorylates AtRGS1 following the perception of glucose and also promotes AtRGS1 endocytosis [14]. The mechanism of 7-TM receptor internalization physically removing the receptor from the cell surface to desensitize cells from continuous stimulation of the G protein complex by the activated receptor is vital for signal transmission balance of living cells [15, 16].

RGS proteins, which accelerate GTP hydrolysis and negatively regulate G protein-coupled signaling pathways, are required for the signaling internalization of GPCRs [17, 18]. *M*. *oryzae* contains eight RGS (regulator of G-protein signaling) and RGS-like proteins (MoRgs1 to MoRgs8) [19] that play shared and distinct functions [20]. Among them, MoRgs7 containing an N-terminal 7-TM, in addition to an RGS-like domain, undergoes endocytosis to regulate G-protein/cAMP signaling required for appressorium formation [20]. Further studies suggested that MoRgs7 mediates the perception of hydrophobic environmental cues to govern the intracellular Gα-cAMP signaling pathway, but how MoRgs7 conveys environment cue signaling remains unclear.

Previous studies showed that MoSep1, a yeast Ste/Ste11/Cdc15 protein kinase homolog, functions as a part of the Mitotic-Exit Network (MEN) that phosphorylates the Cell Wall Integrity (CWI) MAP kinase MoMkk1 indispensable in the development and virulence of *M*. *oryzae* [21]. Intriguingly, we found that MoSep1 also phosphorylates MoRgs7, modulating its binding affinity with the actin-binding Coronin-like protein MoCrn1, and that MoCrn1 is involved in the endocytosis of MoRgs7. Thus, MoSep1 is linked to Gα-cAMP signaling through MoSep1-dependent MoRgs7 phosphorylation and MoCrn1 binding. Our findings revealed a novel mechanism of MoRgs7-mediated hydrophobic cue signal transduction in the appressorium formation and pathogenesis of the blast fungus.

## Results

### MoRgs7 is phosphorylated during appressoria formation

**Fig 1:**
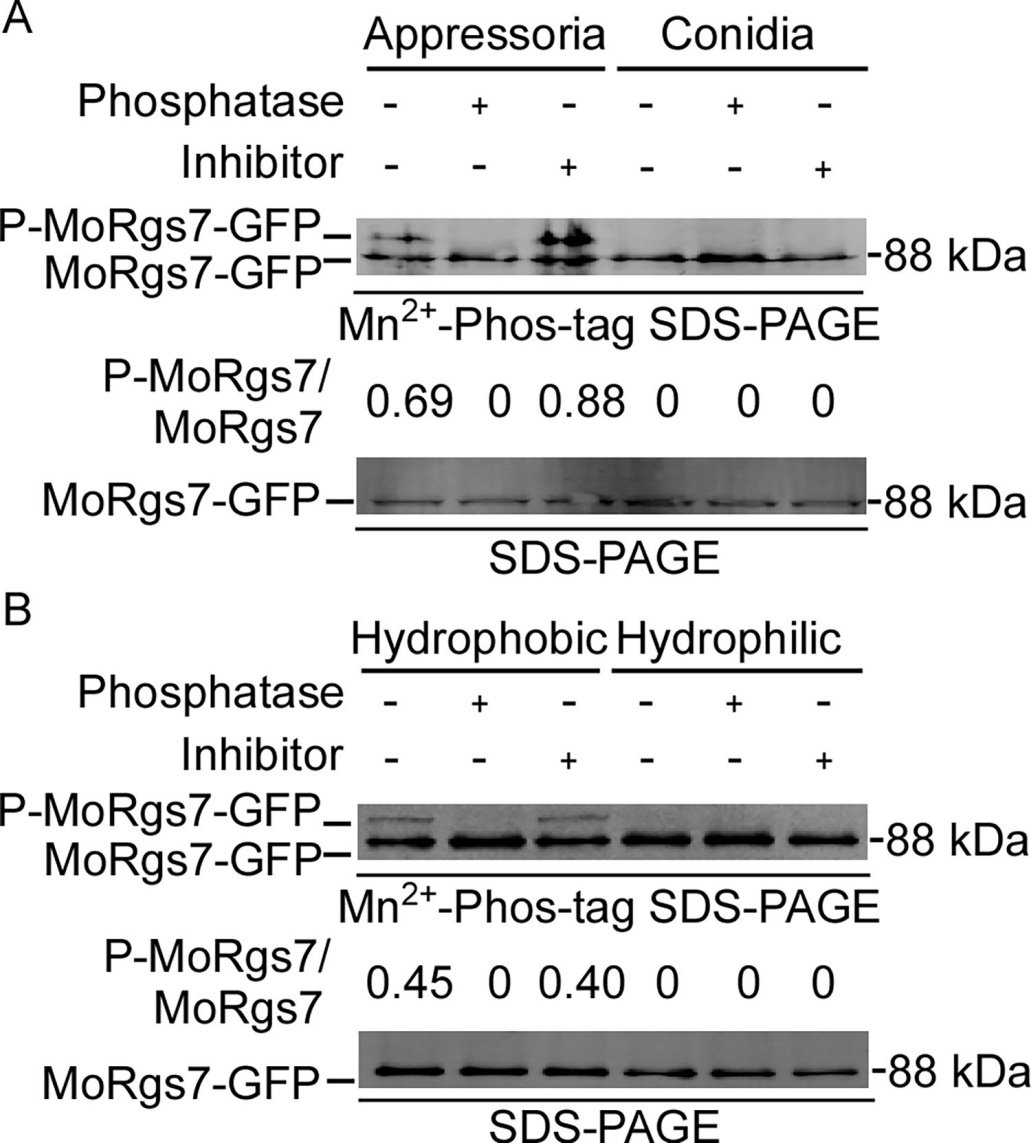
MoRgs7 is phosphorylated during the early appressorial stage of *M*. *oryzae*. (A) MoRgs7-GFP proteins were extracted from transformants at conidia and appressoria stages, then treated with phosphatase and phosphatase inhibitors and shifted by Mn^2+^-Phos-tag SDS-PAGE and normal SDS-PAGE with the anti-GFP antibody. For total protein extraction, 1 mL lysis buffer (10 mM Tris-HCl, pH 7.5, 150 mM NaCl, 0.5 mM EDTA, 0.5% NP-40 [Sigma-Aldrich, I3021]) with 2 mM PMSF (Beyotime Biotechnology, ST506-2), proteinase inhibitor cocktail (Sigma-Aldrich, 11836170001) and deacetylation inhibitors (50 mM nicotinamide, 50 mM sodium butyrate, 5 mM Trichostatin A [Sigma-Aldrich, T1952]) were used to resuspend ground powder of appressoria and conidia. (B) Phosphorylation analysis of MoRgs7-GFP in Guy11 (the steps are similar to A). MoRgs7-GFP proteins were extracted from conidia of transformants which were allowed to germinate on hydrophobic and hydrophilic surfaces at 4 h-post inoculations.

MoRgs7 is important in hydrophobic surface-induced appressorium formation in *M*. *oryzae* [20]. To investigate the underlying relationship between MoRgs7 and hydrophobic surface, we first examined whether MoRgs7 is phosphorylated by comparing conidial and appressorial stages using Mn^2+^-Phos-tag gel electrophoresis. We found that the band representing the phosphorylated-MoRgs7-GFP (P-MoRgs7) was apparent in the appressorial stage but not the conidial stage. The P-MoRgs7 band was absent in the presence of a phosphatase but more prominent in the presence of a phosphatase inhibitor (Fig 1A). To examine whether MoRgs7 phosphorylation is also responsive to hydrophilic cues, we compared proteins extracted from germinated conidia after 4 h of incubation on hydrophobic and hydrophilic surfaces. The result showed that MoRgs7-GFP is phosphorylated only when cultivated on the hydrophobic surface. This agrees with MoRgs7 subject to phosphorylation regulation during the early appressorial stage when recognizing hydrophobic surface cues (Fig 1B).

### MoRgs7 is phosphorylated by MoSep1

**Fig 2:**
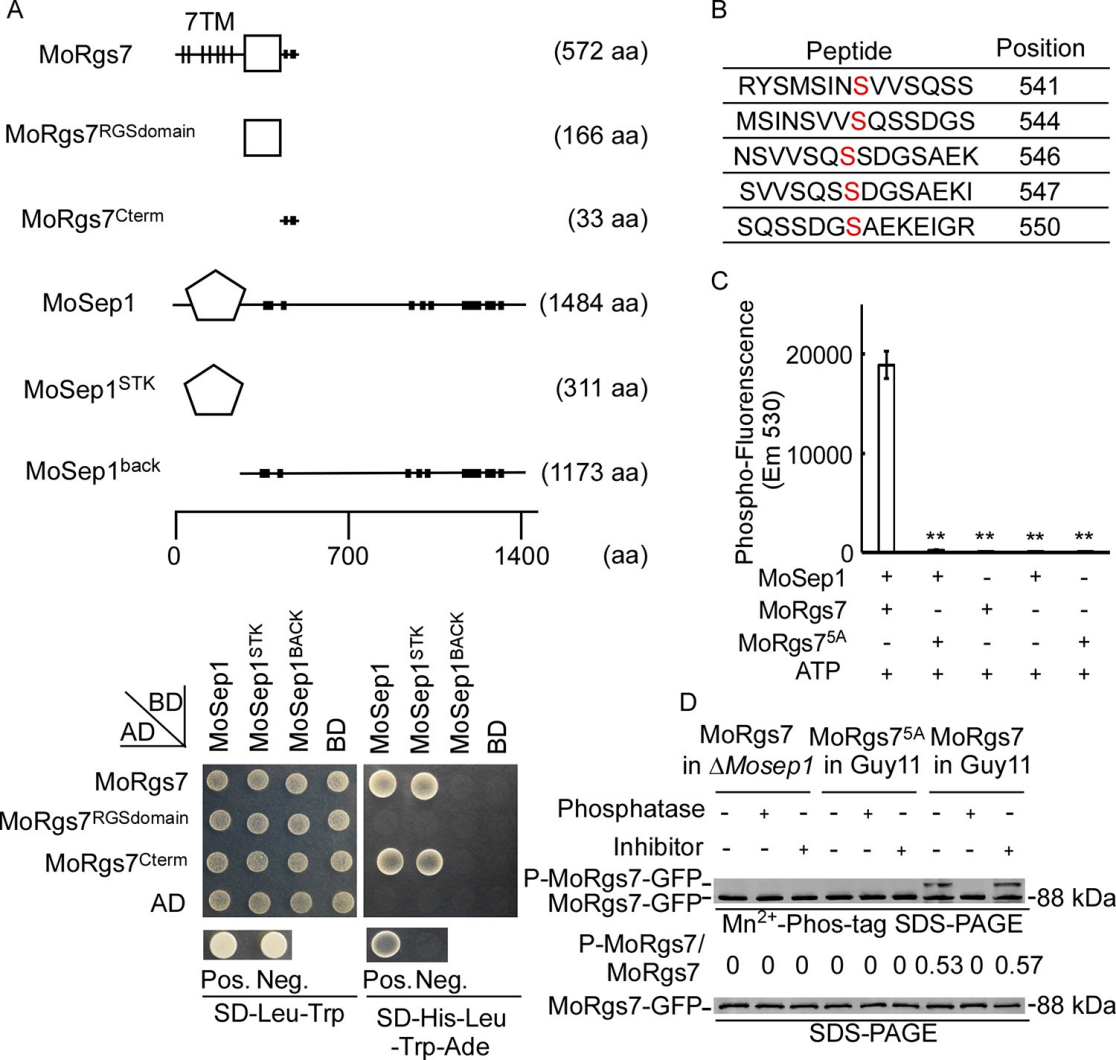
MoSep1 functions as a kinase for MoRgs7 phosphorylation. (A) Schematic representation of MoRgs7 and MoSep1.The RGS domain and S_TKc domain were predicted by the SMART program (http//smart.embl-heidelberg.de/). S_TKc domain was Serine/Threonine protein kinases catalytic domain and had been defined as MoSep1^STK^. The region back of S_TKc domain had no functional domain and had been defined as MoSep1^BACK^. The truncated domains were named similarly to the previous study [21]. aa, amino acid. Yeast two-hybrid analysis. MoRgs7 and its two segments MoRgs7^RGSDomain^ and MoRgs7^Cterm^ were co-introduced with MoSep1, MoSep1^BACK^, and MoSep1^STK^ into the AH109 strain, respectively. The transformants were plated on SD-Leu-Trp and on SD-Leu-Trp-His-Ade for 5 days. (B) Phosphorylation sites were identified by comparing the wild type (Guy11) with the Δ*Mosep1* mutant expressing MoRgs7 in LC-MS-MS (Q-E) analysis. Pos., positive control. Neg., negative control. (C) *In vitro* phosphorylation analysis by the fluorescence detection in tube (FDIT) method. In the presence of 50 μm ATP, the purified proteins of GST-MoRgs7, GST-MoRgs7^5A^, and His-MoSep1 were constructed for protein kinase reactions. The fluorescence signal at 590 nm (excited at 530 nm) was measured in a Cytation3 microplate reader (Biotek, Winooski, VT, USA). Error bars represent SD and asterisks represent significant differences (**p < 0.01). (D) Phosphorylation analysis of MoRgs7 *in vivo*. MoRgs7-GFP from corresponding transformants in appressorial stages was treated with a phosphatase (PE) and a phosphatase inhibitor (PI). Proteins were detected by the GFP antibody and shifted by Mn^2+^-Phos-tag SDS-PAGE and normal SDS-PAGE, respectively.

To identify protein kinases that phosphorylate MoRgs7, we screened a Y2H cDNA library of *M*. *oryzae* using BD-MoRgs7, conducting by cloning full-length cDNAs of MoRgs7 into pGBKT7, as a bait vector and identified MoSep1 (MGG_04100) (S1 Text), a cell cycle-related kinase homolog, as a potential candidate. We further verified the interaction by Y2H and demonstrated that MoRgs7 and MoRgs7^Cterm^ interacts with both MoSep1 and MoSep1^STK^ (Fig 2A). To further examine the MoRgs7 and MoSep1 interaction, we generated two partial clones of MoSep1, MoSep1^STK^, and MoSep1^BACK^, and carried out co-IP and GST-pulldown assays. The assays showed that MoRgs7 interacts with both MoSep1 and MoSep1^STK^ but not MoSep1^BACK^ (S1A and S1B Fig).

To identify MoSep1-dependent phosphorylation sites, we compared LC-MS/MS analysis data between the wild-type (WT) Guy 11 and the Δ*Mosep1* mutant, and identified five differentiated serine phosphorylation sites, including S541, S544, S546, S547, and S550 (Figs 2B and S2). Each individual phosphorylation site was mutated to a negatively charged amino acid, aspartic acid, to mimic phosphorylation status, or to a non-phosphorylatable amino acid, alanine. After checking the phenotype and interaction relationship of each single mutant of MoRgs7, we found that every single mutation affects the MoSep1 and MoRgs7 interaction (S3 Fig). We then performed all five sites Serine (S) to Alanine (A) site-directed mutagenesis to mimic the phospho-dead mutant of MoRgs7 (MoRgs7^5A^), and Serine (S) to Aspartic acid (D) site-directed mutagenesis to mimic the phosphomimetic (MoRgs7^5D^) status. The mutant constructs were linked to either GFP or GST marker proteins. Phosphorylation assays with *in vivo* Mn^2+^-Phos-tag gel analysis and *in vitro* fluorescence detection in tube (FDIT) confirmed the phosphorylation relationship between MoSep1 and MoRgs7 depends on these serine phosphorylation sites (Fig 2C and 2D).

### MoRgs7 phosphorylation is important for pathogenicity

**Fig 3:**
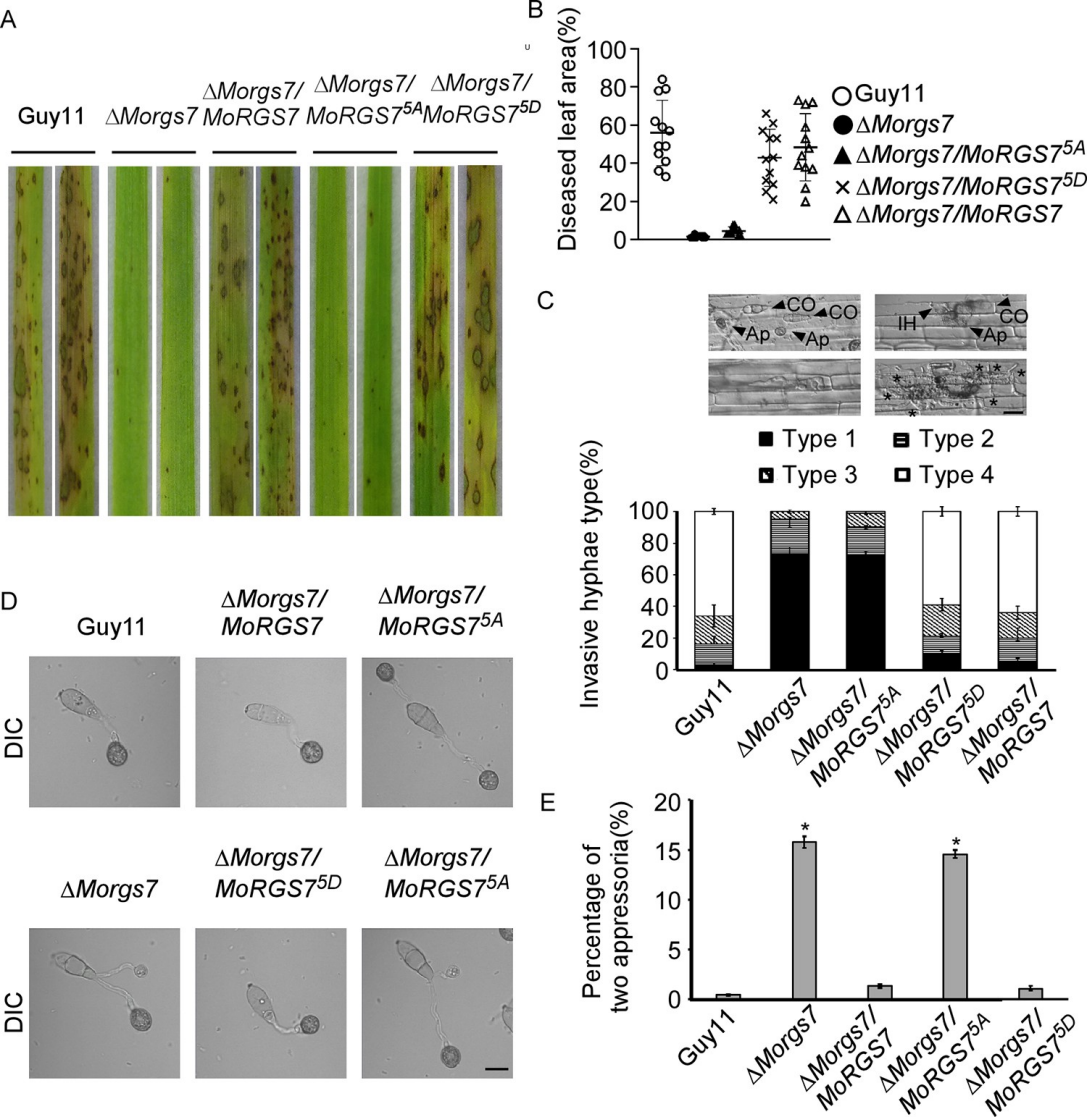
MoSep1-dependent MoRgs7 phosphorylation is required for pathogenicity. (A) Pathogenicity assay was conducted with two-week-old rice seedlings (*Oryza sativa* cv.CO39), which were sprayed by conidial suspensions (5×10^4^ spores/ml) of each strain. After 7 days post-incubation (dpi), diseased leaves were photographed. (B) Diseased leaf areas were evaluated by Image J analysis and scatter plotted. Error bars represent the standard deviations (n = 12). (C) Detailed observation and statistical analysis of infectious hyphal type in rice sheath cells at 36 hpi. (Type 1, appressoria formation with no penetration; type 2, penetration with short IH; type 3, invasive IH extended within a plant cell; type 4, extensive hyphal growth). One hundred infectious hyphae were assessed for each strain. The mean values of three repeated experiments with standard deviations are shown. Scale bar: 10 μm. (D) Appressorium formation assays. The conidia of each strain were photographed after 24 hours of incubation. Scale bar: 10 μm. (E) Percentage of two appressoria, indicating its defect in appressorial development. One hundred conidia of each strain were observed after 24 hours of incubation. Error bars represent SD and asterisks represent significant differences (*p < 0.05).

To examine if MoRgs7 phosphorylation affects fungal pathogenicity, susceptible rice seedlings (CO-39) were sprayed with conidia of the wild-type Guy11, the Δ*Morgs7* mutant, and the complemented Δ*Morgs7/MoRGS7* strains, as well as the site-directed Δ*Morgs7/MoRGS7*^5A^ and Δ*Morgs7/MoRGS7*^5D^ mutant strains. Very few lesions were found in leaves infected with Δ*Morgs7* and Δ*Morgs7/MoRGS7*^5A^ in comparison to Δ*Morgs7/MoRGS7*^5D^ and other strains (Fig 3A and 3B). Appressorial penetration and invasive hyphal growth assays indicated less than 25% appressorial penetration by the Δ*Morgs7* and Δ*Morgs7/MoRGS7*^5A^ strains at 24 hpi (Fig 3C). These results indicated the importance of MoSep1-dependent MoRgs7 phosphorylation in the appressorial formation and virulence of *M*. *oryzae*.

We also compared the proportion of appressorium penetration in onion epidermal cells, and the results indicated that Δ*Morgs7* and Δ*Morgs7/MoRGS7*^5A^ were defective in penetration when compared with Guy11 (S4A Fig). Further tests using various concentrations of glycerol to assess appressorial turgor pressure showed apparent differences. At 1 M of glycerol, the appressorial collapse rate was higher in Δ*Morgs7* and Δ*Morgs7/MoRGS7*^5A^ strains than that in Guy11 and the Δ*Morgs7/MoRGS7*^5D^ strain. Moreover, the proportion of collapsed appressoria in Δ*Morgs7/MoRGS7*^5A^ continuously increased at a faster rate than that in Guy11 and Δ*Morgs7/MoRGS7*^5D^ strains until glycerol reached 4M. These data suggested that MoRgs7 phosphorylation plays a role in maintaining turgor pressure. (S4B Fig).

The effect of MoRgs7 phosphorylation in appressorium formation evokes that the defect of RGS family proteins leads to abnormal perception of hydrophobic cues and could form two appressoria from a single conidium [19]. Examining the effect of MoSep1-dependent MoRgs7 phosphorylation on appressorial development showed that the percentage of conidia forming two appressoria was approximately 15% in the Δ*Morgs7* and Δ*Morgs7/MoRGS7*^5A^ strains, while lower than 5% was recorded in Δ*Morgs7/MoRGS7*^5D^ (Fig 3D and 3E). These results suggested that MoSep1-dependent MoRgs7 phosphorylation is involved in appressoria induction.

It has been reported that MoSep1 is involved in Mitotic Exit Network (MEN) and Cell Wall Integrity (CWI) signaling that directly affects the pathogenicity of *M*. *oryzae* [21]. To examine if MoSep1-dependent MoRgs7 phosphorylation promotes MoSep1 function in MEN and CWI signaling, we expressed MoRgs7^5D^-GFP constructs into *ΔMosep1* and examined its vegetative growth, conidiation, and virulence. The results showed that continuous phosphorylation of MoRgs7 cannot restore the growth and virulence defect of Δ*Mosep1* (S5 Fig).

### MoRgs7 phosphorylation is involved in MoMagA-mediated cAMP signaling and normal appressorial induction

**Fig 4:**
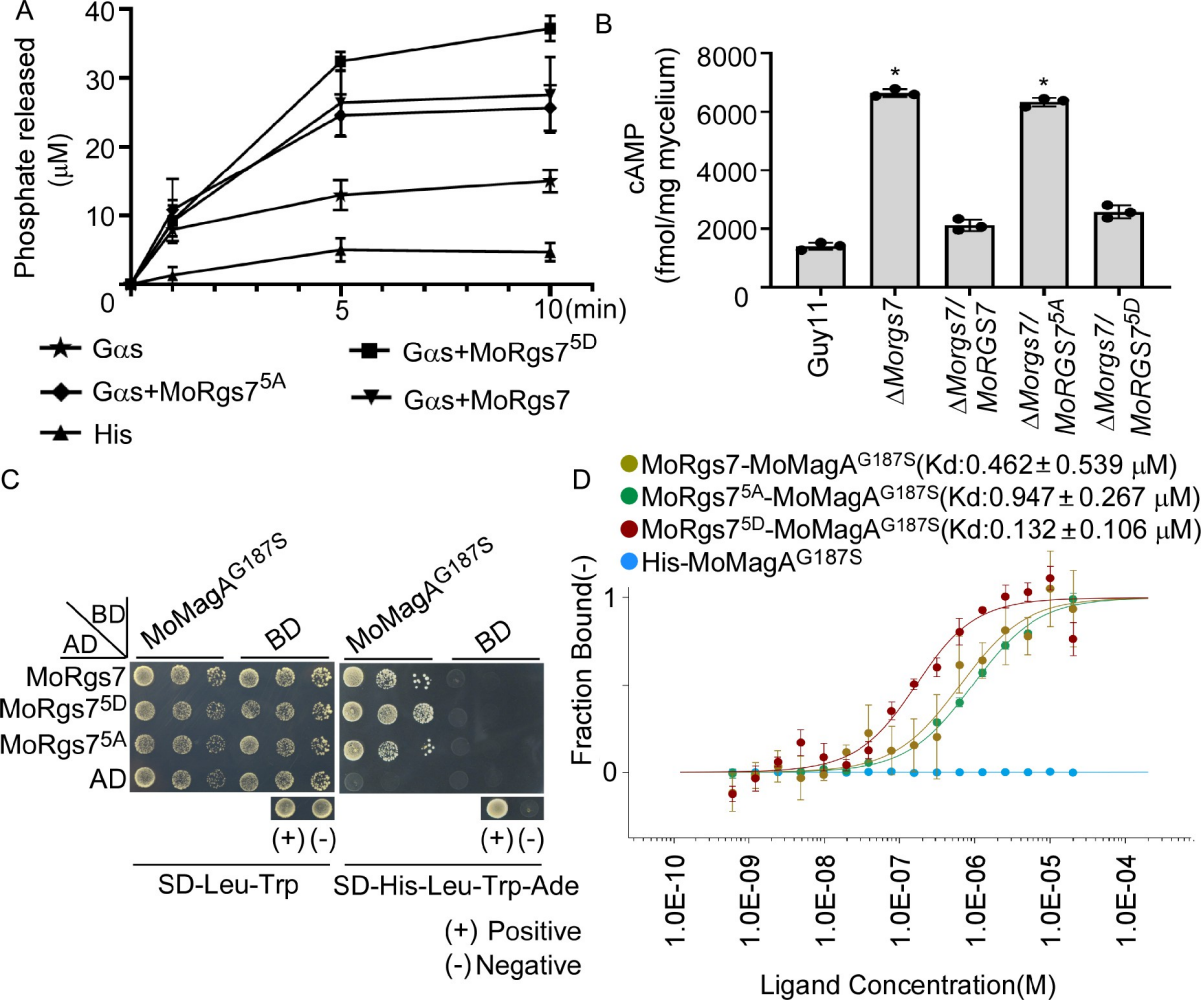
MoSep1-dependent MoRgs7 phosphorylation is required for cAMP signaling. (A) Measurement of GTPase rates. Free phosphates liberated by enzymes were measured using a GTPase activity kit. MoRgs7 and its site-directed mutagenesis MoRgs17^5A^ and MoRgs7^5D^ were measured at least 3 times. Error bars indicate SDs. (B) Measurement of intracellular cAMP levels in mycelia. The levels of cAMP following 2 days of culturing in the complete medium (CM) are shown. The data is evaluated by HPLC analysis with three replicates. Error bars represent SD and asterisks represent significant differences (*p < 0.05). (C) Yeast two-hybrid analysis. MoMagA^G187S^ (activated Gα) was co-introduced with MoRgs7 and its site-directed mutagenesis MoRgs7^5A^ and MoRgs7^5D^ into yeast AH109 strain, respectively. Transformants were plated on SD-Leu-Trp and on SD-Leu-Trp-His-Ade for 5 days. (D) MST showing binding properties of MoRgs7 with MoMagA^G187S^.Sorting by Kd value, MoRgs7^5D^<MoRgs7<MoRgs7^5A^. Kd, dissociation constant.

MoRgs7 is one of the RGS family proteins that also plays an indispensable role in cAMP signaling in *M*. *oryzae* [19, 20, 22]. To examine if MoSep1-dependent phosphorylation affects the RGS function of MoRgs7, we assessed *in vitro* GTPase accelerating protein (GAP) activities of MoRgs7, MoRgs7^5A^, and MoRgs7^5D^. The result revealed that the activated MoRgs7^5D^ mutant exhibited the highest GAP activities, while the unphosphorylated MoRgs7^5A^ mutant showed the lowest GAP activities. This finding suggested that MoRgs7 phosphorylation is required for its GAP function (Fig 4A).

The rate of GTPase activity directly affects intracellular cAMP levels. An examination revealed high intracellular cAMP levels in Δ*Morgs7* and Δ*Morgs7/MoRGS7*^*5A*^ than that in Guy 11 and the Δ*Morgs7/MoRGS7* ^*5D*^ complemented strain (Fig 4B). MoRgs7 was previously found to interact only with Gα MoMagA [19, 20, 22]. MoMagA is one of the three Gα subunits (MoMagA, MoMagB, and MoMagC) and plays a major role in being part of Gα subunits that activates the downstream cAMP pathway [19, 23–25]. We generated BD-MoMagA^G187S^ and MoMagA^G187S^-His constructs that mimic GTP-bound Gα [24] to examine if changes in GTPase accelerating protein activities involve affinity bindings between GTP-bound MoMagA and MoRgs7. Y2H, *in vivo* co-IP, and microscale thermophoresis (MST) assays all showed that MoMagA^G187S^ bound with MoRgs7, with the highest affinity being with MoRgs7^5D^ (Figs 4C, 4D, S6A and S6B). In summary, MoSep1-dependent MoRgs7 phosphorylation directly enhances cAMP signaling by exhibiting GAP activities towards MoMagA in *M*. *oryzae*.

### Phosphorylation is vital for MoRgs7 internalization

**Fig 5:**
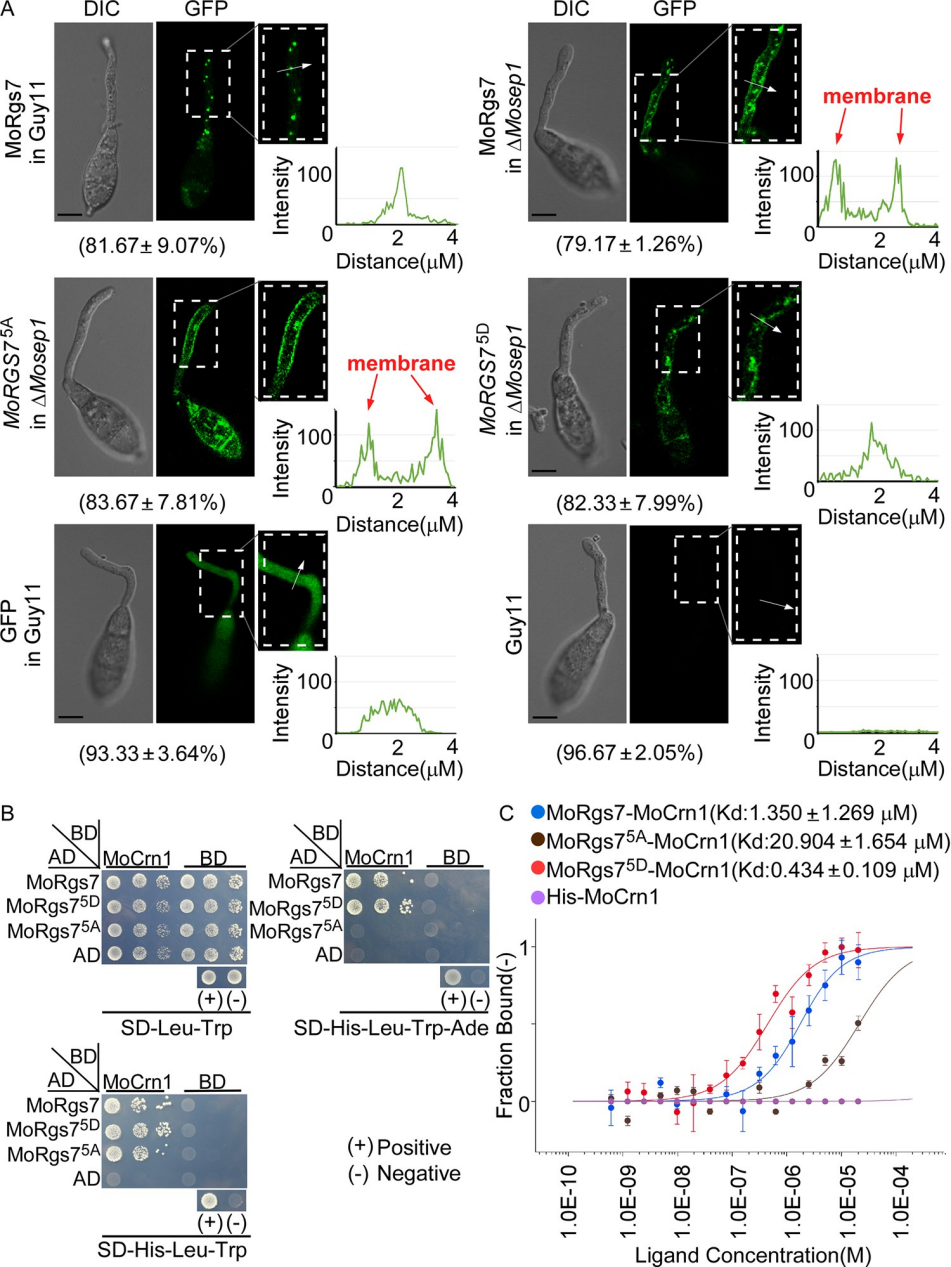
MoSep1-dependent MoRgs7 phosphorylation is important for MoRgs7 endocytosis. (A) The signal of MoRgs7-GFP was enhanced at the PM of germ tubes on the hydrophobic surface at 3 hpi when MoRgs7 was sustainably unphosphorylated. GFP in Guy11 and Guy11 were included as negative controls. The magnified region pointed by white arrows was conducted with line scan analysis. Percentage of a pattern showed in image was calculated by observation for 100 germinated conidia that were randomly chosen. This experiment was performed with three biological replicates. Scale bar: 10 μm. (B) Yeast two-hybrid analysis. MoCrn1 was co-introduced with MoRgs7 and its site-directed mutagenesis MoRgs7^5A^ and MoRgs7^5D^ into the AH109 strain, respectively. Transformants were plated on SD-Leu-Trp (as control), SD-His-Leu-Trp (for initial selection), and SD-Leu-Trp-His-Ade (for further selection) for 5 days. (C) MST showing binding properties of MoRgs7 with MoCrn1. Sorting by Kd value, MoRgs7^5D^<MoRgs7<MoRgs7^5A^. Kd, dissociation constant.

We have previously found that MoRgs7 couples with the actin-binding protein MoCrn1 to undergo endocytosis [20]. We then tested the hypothesis that MoSep1-dependent MoRgs7 phosphorylation also involves actin-dependent endocytosis. Using GFP fusion protein constructs, we found that MoRgs7^5A^-GFP fluorescence was enhanced at the plasma membrane (PM), similar to MoRgs7-GFP in the *ΔMosep1* mutant but in contrast to MoRgs7^5D^-GFP, indicating that MoRgs7 internalization is dependent on MoSep1 (Fig 5A).

Previous studies also indicated that a coronin-like actin-binding protein, MoCrn1, directs MoRgs7 for endocytosis upon sensing hydrophobic cues [20]. To test if MoSep1-dependent MoRgs7 phosphorylation affects its binding affinity with MoCrn1, we assessed the interactions between various constructs (MoRgs7, MoRgs7^5A^, and MoRgs7^5D^) and MoCrn1 by Y2H, *in vivo* co-IP, and MST assays (Figs 5B, 5C, S7A and S7B). The results demonstrated that the balance of MoRgs7 phosphorylation is critical for its binding with MoCrn1. The sustainable phosphomimetic MoRgs7 (MoRgs7^5D^) had a high affinity for MoCrn1, while the sustainable unphosphorylated MoRgs7 (MoRgs7^5A^) interacted weakly with MoCrn1. The above results demonstrated that MoSep1-dependent MoRgs7 phosphorylation enhances the bind of MoRgs7 to MoCrn1, which regulates MoRgs7 internalization.

### Phosphorylation of MoRgs7 promotes its degradation via the ubiquitin-proteasome pathway (UPP)

**Fig 6:**
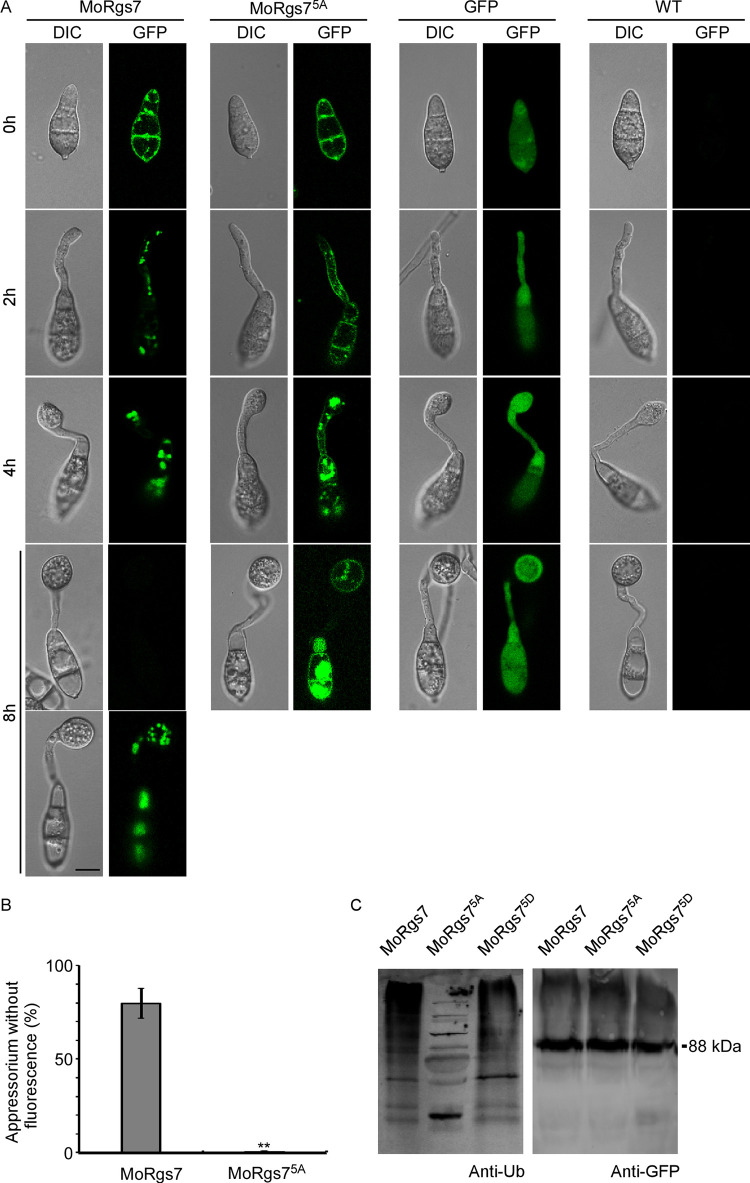
Phosphorylation of MoRgs7 is required for its ubiquitin-mediated protein degradation in the cytoplasm. (A) For MoRgs7-GFP observations, conidia were inoculated on plastic coverslips and incubated in a moist chamber. DIC and epifluorescence images were captured at the indicated time points. Parts of the appressorium showed no GFP signals at 8 hpi for MoRgs7-GFP, in contrast to the strong MoRgs7^5A^-GFP signal in most of the appressoria at the same time. GFP in Guy11 and WT (Guy11) were included as negative controls. Scale bar: 10 μm. (B) One hundred appressoria were quantified for fluorescence intensities. The fluorescence signal assessed using ImageJ. Error bars represent the standard deviations, and asterisks denote statistical significance (**p < 0.01). (C) *In vivo* ubiquitination analysis of protein extracted from MoRgs7, MoRgs7^5A^, and MoRgs7^5D^ transformants and purified with GFP-Sepharose.

Previous studies suggested that MoRgs7 may undergo protein degradation following appressorium maturation and that the degradation is linked to UPP [26]. Monitoring the fluorescence signal with the same setting and of MoRgs7-GFP and MoRgs7^5A^-GFP during appressorium maturation, we found that MoRgs7-GFP fluorescence disappears in approximately 80% of appressoria at 8 hpi, in contrast to MoRgs7^5A^-GFP, whose fluorescence remained at the PM at the same time (Fig 6A and 6B). To further examine whether the disappearance of MoRgs7 involves UPP, we utilized the anti-ubiquitin (Ub) antibody to detect the ubiquitin chain of *in vivo*-purified proteins. After determining the sample loading of eluted proteins quantitatively by anti-GFP, MoRgs7 and MoRgs7^5D^ were more intensively stained with the anti-Ub antibody than MoRgs7^5A^, suggesting that MoRgs7 degradation involves functions of UPP (Fig 6C).

## Discussion

*M*. *oryzae* is the causal agent of rice blast, infecting its host by forming the appressorium that penetrates the host cell. The rice surface builds the primary barrier for host–pathogen interactions, and the characterization of rice surface cues, including hydrophobicity and hardness, is crucial for the early stages of appressorium formation [6]. It is believed that the pathogen surface receptor proteins such as GPCRs sense and internalize environmental cues to activate downstream effectors leading to large-scale gene expression reprogramming resulting in new development processes such as appressorium formation [27]. Receptor internalization undergoes a series of strict regulations, and, while still partly unclear, the regulation involves post-translational modifications (PTMs), including the phosphorylation of intracellular residues that regulate GPCR activities [28]. Our previous studies revealed that the RGS-like domain- and 7-TM domain containing MoRgs7 senses hydrophobic environmental cues and regulates cAMP signaling through MoCrn1-mediated endocytosis [20]. Here, we further demonstrated that MoRgs7 is phosphorylated by MoSep1 in response to hydrophobic surface cues to undergo the endocytic process and that MoSep1-dependent MoRgs7 phosphorylation and endocytosis are critical for the appressorial development and pathogenicity of *M*. *oryzae*.

RGS and RGS-like proteins are directly linked to the Gα-cAMP signaling pathway, which works as negative regulators to enhance intrinsic GTPase activities of GTP-bound Gα subunits, thereby inactivating G protein function [29, 30]. Various studies explore the unique function of RGS proteins by focusing on their phosphorylation. For example, the *S. cerevisiae* RGS protein Sst2 is regulated by a phosphorylation feedback loop involving the MAP kinase Fus3 in response to pheromone stimulation [31]. In *M*. *oryzae*, the phosphorylation of RGS proteins also plays a significant role in fungal development. For example, MoRgs1 phosphorylation by the casein kinase 2 MoCk2 is required for appressorium formation and pathogenicity [32]. Our current studies provided compelling evidence to indicate that MoRgs7 is phosphorylated by the cell cycle-related kinase MoSep1 in response to hydrophobic surface cues, and the phosphorylation and endocytic transport links signaling transmission to fungal pathogenicity.

To establish and maintain a signal transmission balance of cell, cell membrane receptor proteins are redistributed from the PM to cytosol. This dynamic process is vital for GPCR-regulated development processes [33]. Previous studies focused on the process of GPCR trafficking as a way to understand their functional mechanisms. *A*. *thaliana* AtRGS1 endocytosis is regulated by AtWNK8-mediated phosphorylation that sustains environmental sugar signaling [14]. The endocytosis of yeast Ste2p requires the recruitment of Ste2p to preexisting clathrin-coated pits (CCPs). This recruitment is regulated by receptor phosphorylation and subsequent ubiquitination [34]. In *M*. *oryzae*. we have previously reported that the actin-binding protein MoCrn1 directs MoRgs7 to endocytic pits/vesicles for GPCR internalization [20]. Here, we observed that MoCrn1 displays a higher affinity with phosphorylated MoRgs7, suggesting that MoSep1-dependent MoRgs7 phosphorylation is the key to MoCrn1-mediated MoRgs7 internalization. By revealing protein phosphorylation in GPCR endocytosis as a regulatory mechanism of GPCRs, our studies further revealed the complex and multitude of signaling transduction in *M*. *oryzae*.

MoSep1 functions as a Ser-Thr kinase necessary for septum formation and also links the MEN pathway to CWI signaling [21, 35]. Our current studies revealing the additional role of MoSep1 indicate MoSep1 as a protein kinase of many different functions. Intriguingly, the phosphomimetic MoRgs7 suppressed the defect of appressorium formation in the Δ*Morgs7* mutant but not the virulence defect of Δ*Mosep1*, suggesting that MoSep1 mediated MoRgs7 phosphorylation is independent of its regulatory mechanisms in MEN and CWI pathways (S8A and S8B Fig).

**Fig 7:**
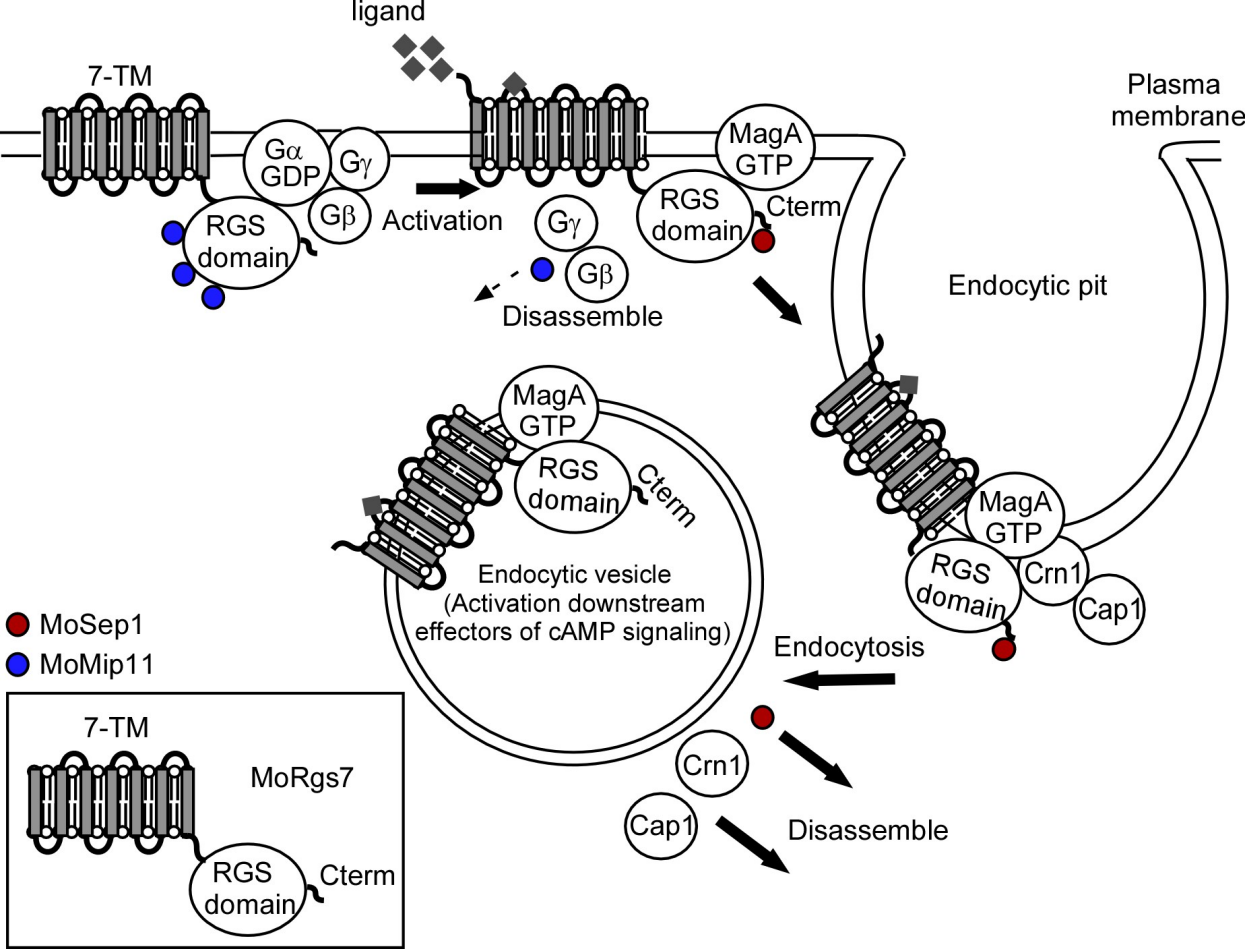
A schematic summary of MoRgs7 function during appressorium formation in *M*. *oryzae*. MoSep1-dependent MoRgs7 phosphorylation promotes hydrophobic signaling internalization through MoCrn1-mediated endocytosis. In response to hydrophobic surface cues, MoRgs7 becomes phosphorylated by the cell cycle-related kinase MoSep1. MoRgs7 phosphorylation promotes its internalization through MoCrn1-mediated endocytosis and interaction with MoMagA^G187S^ (the activated Gα) that regulates intracellular cAMP levels. When the interaction between MoRgs7 and MoSep1 is blocked, the defect in transducing hydrophobic signals results in abnormal appressorium formation and attenuation in pathogenicity.

In summary, we have identified MoRgs7 phosphorylation by MoSep1 as a novel functional mechanism of MoRgs7 in regulating hydrophobicity-induced cAMP signaling in *M*. *oryzae*. MoRgs7 contained a GPCR-like 7-TM motif in addition to its RGS-like domain and was identified to function in sensing hydrophobic environmental cues [8, 20, 36]. We have demonstrated that MoRgs7 phosphorylation by MoSep1 is required for signal transmission through MoCrn1-dependent endocytosis and the ubiquitin proteasome pathway. In addition, MoSep1-dependent MoRgs7 phosphorylation regulates intracellular cAMP levels for normal appressorium formation and pathogenicity of *M*. *oryzae*. Our studies promote a further understanding of pathogenesis mechanisms during rice blasts and benefit the discovery of novel disease management strategies (Fig 7).

## Materials and methods

### Strains and cultural conditions

Guy11 was used as the wild-type strain of *M*. *oryzae* and cultured on a complete medium (CM). For vegetative growth, small mycelium blocks from 5-day-old colonies were transferred into fresh media and incubated at dark 28°C. Mycelia were harvested from liquid CM medium grown for 2 days and used for DNA and protein extractions [37, 38]. The wild-type and mutants were cultured on an SDC medium at 28°C for 3 days in the dark and transferred to continuous illumination under fluorescent before being scraped for surface hyphae and conidia evaluation [26].

### Protein extraction and Western blot analysis

For total protein extraction, strains were incubated in liquid CM media with shaking for 2 days and harvested. Mycelia were grounded into fine powder in liquid nitrogen and suspended in lysis buffer (10 mM Tris-HCl, pH 7.5, 150 mM NaCl, 0.5mM EDTA, 0.5% NP-40, and 2 mM PMSF). The lysates were collected into 2.0 ml tubes in ice for 30 min and shaken every 10 min. Lysates were then centrifuged at 15,000 rpm for 10 min at 4°C and supernatants were collected as total protein extracts [39]. For GFP-tagged protein detection, samples were analyzed with 12% SDS-PAGE gel and immunoblotted with anti-GFP antibodies (mouse, 1:5000, Abmart, 293967). Signals were detected by the ODYSSEY infrared imaging system (software Version 2.1). For ubiquitin chain detection, samples were analyzed using the anti-Ub antibody (mouse, 1:5000, ptmbiolabs, PTM-1107) [40].

### Phosphorylation analysis

For *in vitro* analysis, GST-MoRgs7, GST-MoRgs7^5A^, and His-MoSep1 were expressed in *E*. *coli* DE3 cells and purified [41]. We used the Pro-Q Diamond Phosphorylation gel stain (Thermo Fisher Scientific), a phosphor-protein gel-staining fluorescence dye in this assay. A kinase reaction buffer (100 mM phosphate-buffered saline, pH 7.5, 10 mM MgCl_2_, 1 mM ascorbic acid) was mixed with MoRgs7 and His-MoSep1, MoRgs7^5A^, and His-MoSep1, respectively. The subsequent experiments were performed according to the previously described protocol [21].

For *in vivo* analysis, conidia were prepared from various transformants as described above and were filtered through three layers of lens paper before resuspending in sterile water (2 ×10^5^ spores mL^-1^) [42]. For appressorium protein extraction, droplets (5 mL) of spore suspensions were placed on strips of onion epidermis, incubated under humid conditions at room temperature for 6 h, and onion epidermis grounded for protein extraction [43]. Protein extraction was the same as described above and phosphorylation analysis was performed as according to the protocol, phosphatase inhibitors (P0044, sigma) and alkaline phosphatase (P6774, sigma) [21].

### Virulence test

Rice seedlings (*Oryza sativa* cv. CO39) were used for the pathogenicity test. Two-week-old rice seedlings were sprayed with 5 mL of conidia suspension and kept in darkness for 24 hours. Then rice seedlings were transferred into a transparent growth chamber following a 12 h/12 h light/dark cycle exposure schedule. After seven days, leaf disease severity was documented by photography. Lesions were quantitated by ImageJ [44].

3-week-old rice sheaths were collected and injected with conidia suspensions (2 × 10^5^ spores/ml) with syringes. After 36 hours post-incubation at dark 28°C, epidermal cells were observed by microscopy. This experiment was conducted with three biological repetitions and three replicates under the same experimental conditions [45].

### LC-MS-MS analysis and GTPase activity assays

Fungal proteins were extracted as described above and separated by 10% SDS-PAGE gel electrophoresis. Phosphorylation site identification by LC-MS-MS was carried out as described previously [21].

His-MoRgs7, His-MoRgs7^5A^, His-MoRgs7^5D^, and His-MoMagA^G187S^ were expressed in *E*. *coli* DE3 cells and purified. An ATPase/GTPase Activity Assay kit (MAK113; Sigma-Aldrich; Merck, Darmstadt, Germany) was used to assess the GTPase activity. The phosphate standards included in the kit were used to plot the standard curve. Reactions were incubated for 0 s, 1 min, 5 min, and 10 min at room temperature. Reagent buffer (200 ml) was added to each well, and incubation was carried out for an additional 30 mins at room temperature to terminate the enzyme reaction and generate the colorimetric end product. Absorbance at 620 nm was read and the change in absorbance values (DA620) was calculated by subtracting the control well (A620) from the sample well (A620). The concentration (μM) of free phosphate [Pi] was computed in the sample from the standard curve. The relevant experimental procedures were as described previously [32].

### Intracellular cAMP level measurement

*M*. *oryzae* strains and transformants were cultured in liquid CM for 48 h and harvested. Mycelia were grounded into fine powders in liquid nitrogen and total cAMP levels were quantified by HPLC following previously established procedures [32].

### Microscale thermophoresis assay

Bindings of MoCrn1 to MoRgs7, MoRgs7^5A^, MoRgs7^5D^, and MagA^G187S^ to MoRgs7, MoRgs7^5A^, and MoRgs7^5D^ were determined by MST using Monolith NT.115 (Nano Temper Technologies) according to the manufacture provided protocol. MST premium-coated capillaries (Monolith NT.115 MO-K005) were used to load the samples into the MST instrument at 25°C using medium MST power. Laser on and off was set at 30 and 5 s, respectively. This experiment was repeated with three biological repetitions and three replicates under the same experimental conditions. Data were analyzed using Nano Temper Analysis software v. 1.2.101 (Nano Temper Technologies) [40].

### GST pull-down assay

GST-MoRgs7, His-MoSep1, His-MoSep1^back^, His-MoSep1^STK^, and His were expressed in *E*. *coli* BL21-CodonPlus (DE3) cells. Samples were induced with 0.1 mM IPTG (isopropyl-b-D-1-thiogalactopyranoside) at 20°C for 4 h. *E*. *coli* Cells were lysed in lysis buffer (50 mM Tris [pH = 8.0], 50 mM NaCl, 1 mM phenylmethylsulfonylfluoride [PMSF] [Sigma Aldrich]). Samples were centrifuged at 3,600 rpm for 10 min, and the supernatants were transferred to new 1.5-mL tubes and stored at -70°C. The GST-MoRgs7 supernatant was mixed with 30 mL of GSH Sepharose beads, incubated at 4°C for 4 h, and then incubated with His-MoSep1, His-MoSep1^back^, His-MoSep1^STK^, and His supernatants at 4°C for another 4 h. Finally, the beads were washed with buffer (50 mM Tris, pH 8.0, 50 mM NaCl, 1 mM PMSF, 1% Triton X-100) 5 times and proteins eluted. Eluted proteins were analyzed by immunoblot (IB) with anti-His and anti-GST antibodies [46].

### Confocal laser scanning microscopy

All transformants were observed under a confocal laser scanning microscope (Zeiss LSM710, 63 × oil), and the filtered channels were set as followings: GFP (excitation spectra: 488 nm, emission spectra: 510 nm, intensity of fluorescence: 75%). Exposure time: 800 ms. Insets highlight areas analyzed by line-scan. Bar = 10 μm.

### Appressorium formation and turgor pressure estimation

Conidia were harvested from 7-day-old SDC cultures and adjusted to 5×10^4^ conidia ml^-1^ in sterile water. Droplets (30 μl) of conidial suspension were applied on coverslips (Fisher Scientific, St Louis, MO) and incubated under humid conditions at 28°C prior to appressorial formation assessment [47].

The turgor pressure of mature appressorium was measured using an incipient cytorrhysis (cell collapse) assay. The 1.0–4.0 M glycerol solution was used to measure turgor pressure according to the previous description [21]. Collapsed appressoria were calculated based on data from at least 100 appressoria. This data had three replicates [21].

### Statistical analysis

Results are presented as the mean ± standard deviation (SD) of at least three replicates. Significant differences between replicates were statistically evaluated using SDs and one-way ANOVA in SPSS 2.0. Results of two different treatments were statistically compared by ANOVA, followed by the F-test if the ANOVA result was significant at a *p* value of <0.05 or <0.01.

## Supporting information

S1 Fig MoRgs7 interacts with MoSep1 and MoSep1^STK^. (A) *In vitro* pull-down assay examines interactions between MoRgs7 and MoSep1 full-length, and MoRgs7 and two segments of MoSep1 (MoSep1^STK^ and MoSep1^BACK^). His-MoSep1, His-MoSep1^STK^, and His-MoSep1^BACK^ were expressed and incubated with Sepharose beads. Eluted proteins were detected with anti-His and anti-GST antibodies and analyzed by immunoblotting. Asterisks indicate the main bands. (B) Co-IP assay for the interaction between MoRgs7 and MoSep1 full-length, and MoRgs7 and two segments of MoSep1 (MoSep1^STK^ and MoSep1^BACK^). Co-expression of MoRgs7-RFP and MoSep1^STK^-GFP, MoRgs7-RFP, and MoSep1^BACK^-GFP in the wild-type strain Guy11. The proteins were incubated with anti-RFP beads and detected by anti-GFP and anti-RFP antibodies, respectively. Asterisks indicate the main bands.
(TIF)

S2 Fig MoSep1-dependent MoRgs7 phosphorylation sites identified by LC-MS-MS (Q-E) analysis. MoRgs7 phosphorylation sites in Guy11 in comparison with the Δ*Mosep1* mutant expressing MoRgs7 variants.
(TIF)

S3 Fig The phenotype and interaction relationship of each single mutants of MoRgs7. (A) Pathogenicity assay. The experimental procedures were conducted as the same as described for Fig 3. (B) Yeast two-hybrid analysis. MoSep1 was co-introduced with MoRgs7 and its each site-directed mutant into the AH109 strain, respectively. Transformants were plated on SD-Leu-Trp (as control) and SD-Leu-Trp-His-Ade (for further selection) for 5 days.
(TIF)

S4 Fig MoSep1-dependent MoRgs7 phosphorylation is required for appressorium function. (A) The cytorrhysis assay was conducted by incubating conidia on the hydrophobic surface and treated with various concentrations of glycerol (1–4 M). Numbers of collapsed appressoria among 100 appressoria were recorded and the examination was repeated twice. Error bars represent SDs and asterisks represent significant differences (**p < 0.01). (B) Observation for penetration on the rice sheath. The penetration rate was counted 3 times. Error bars represent SDs and asterisks represent significant differences (*p < 0.05).
(TIF)

S5 Fig Continuous phosphomimetic MoRgs7 fails to restore the defect of MoSep1 in growth and virulence. (A, B, and C) Pathogenicity assay, diseased leaf area analysis, and infectious hyphal type assessment were conducted as the same as described for Fig 3. (D) Statistical analysis of conidia. Conidia grown on SDC medium for 7 days in the dark followed by 3 days of continuous fluorescence illumination at 28°C were assessed. Error bars represent SD and asterisks represent significant differences (**p < 0.01).
(TIF)

S6 Fig MoMagA^G187S^ interacts with MoRgs7, MoRgs7^5A^, and MoRgs7^5D^. (A) Yeast two-hybrid analysis. MoMagA^G187S^ was co-introduced with MoRgs7 and the site-directed mutagenesis mutants MoRgs7^5A^ and MoRgs7^5D^ into the AH109 strain. The transformants were plated on SD-Leu-Trp (as control), SD-His-Leu-Trp (for initial selection), and SD-Leu-Trp-His-Ade (for further selection) for 5 days. (B) Co-IP assay for the interaction between MoMagA^G187S^, MoRgs7 and its site-directed mutagenesis MoRgs7^5A^ and MoRgs7^5D^. Co-expression of MoMagA^G187S^-RFP and MoRgs7-GFP, MoRgs7^5A^-GFP and MoRgs7^5D^-GFP in Guy11, respectively. Proteins were incubated with anti-RFP beads and detected by anti-GFP and anti-RFP antibodies.
(TIF)

S7 Fig MoCrn1 interacts with MoRgs7, MoRgs7^5A^, and MoRgs7^5D^. (A) Yeast two-hybrid analysis. MoCrn1 was co-introduced with MoRgs7 and its site-directed mutagenesis MoRgs17^5A^ and MoRgs7^5D^ variants into AH109 strain, respectively. Transformants were plated on SD-Leu-Trp (as control), SD-His-Leu-Trp (for initial selection), and SD-Leu-Trp-His-Ade (for further selection) for 5 days. (B) Co-IP assay for the interaction between MoCrn1, MoRgs7 and its site-directed mutagenesis MoRgs7^5A^ and MoRgs7^5D^. Co-expression of MoCrn1-RFP and MoRgs7-GFP, MoRgs7^5A^-GFP and MoRgs7^5D^-GFP in the wild-type strain Guy11, respectively. Proteins were incubated with anti-RFP beads and detected by anti-GFP and anti-RFP antibodies.
(TIF)

S8 Fig MoSep1-dependent MoRgs7 phosphorylation is independent of CWI and MEN signaling in *M*. *oryzae*. (A) Hyphae of Guy11 and the ΔMosep1, Δ*Mosep1/MoSEP1*, and Δ*Mosep1/MoRGS7*^5D^ mutants expressing the H1-RFP construct were stained with CFW and examined by epifluorescence microscopy. Scale bar: 10 μm. (B) Hyphae of Guy11 and the Δ*Mosep1*, Δ*Mosep1/MoSEP1*, and Δ*Mosep1/MoRGS7*^5D^ mutants were stained with CFW and examined by epifluorescence microscopy. Percentages shown in all images were calculated by the observation of 100 randomly-selected hyphae, and the observation was repeated twice. Scale bar: 10 μm.
(TIF)

S1 Text Identification of MoRgs7 binding proteins. MoRgs7 was inserted into vector pGBKT7, and was were co-introduced with a yeast two-hybrid cDNA library contained various stages RNS of *Magnaporthe oryzae* into yeast AH109 strain. Then, AH109 strain was incubated on SD-Leu-Trp and SD-Leu-Trp-His-Ade for 5 days. The names of known protein filled in the brackets. The underlined protein is mentioned in this research. Among this table, MoMagA (MGG_01818) and MoCrn1 (MGG_06389) has been proved been interacted with MoRgs7 in previous studies (Li et al., 2019b; Zhang et al., 2011b).
(DOCX)
