# Supplementary figures and images for: Hydrophobic cue-induced appressorium formation depends on MoSep1-mediated MoRgs7 phosphorylation and internalization in Magnaporthe oryzae
Source: PLoS Genet. 2023 May 15;19(5):e1010748. doi: 10.1371/journal.pgen.1010748 (PMC10184898; doi:10.1371/journal.pgen.1010748)

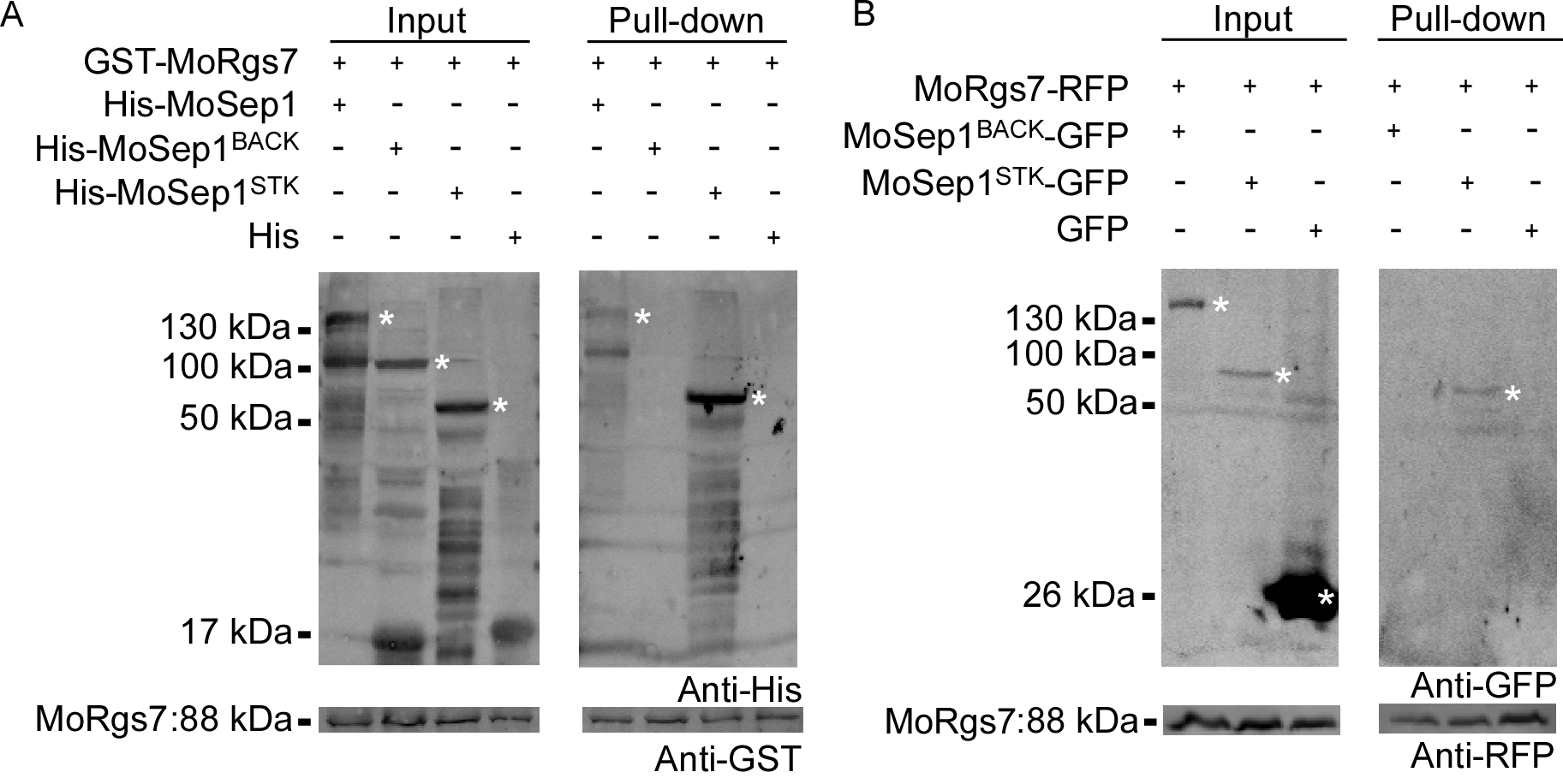

Supplement: S1 Fig — (A) In vitro pull-down assay examines interactions between MoRgs7 and MoSep1 full-length, and MoRgs7 and two segments of MoSep1 (MoSep1STK and MoSep1BACK). His-MoSep1, His-MoSep1STK, and His-MoSep1BACK were expressed and incubated with Sepharose beads. Eluted proteins were detected with anti-His and anti-GST antibodies and analyzed by immunoblotting. Asterisks indicate the main bands. (B) Co-IP assay for the interaction between MoRgs7 and MoSep1 full-length, and MoRgs7 and two segments of MoSep1 (MoSep1STK and MoSep1BACK). Co-expression of MoRgs7-RFP and MoSep1STK-GFP, MoRgs7-RFP, and MoSep1BACK-GFP in the wild-type strain Guy11. The proteins were incubated with anti-RFP beads and detected by anti-GFP and anti-RFP antibodies, respectively. Asterisks indicate the main bands. (TIF) [file pgen.1010748.s001.tif]

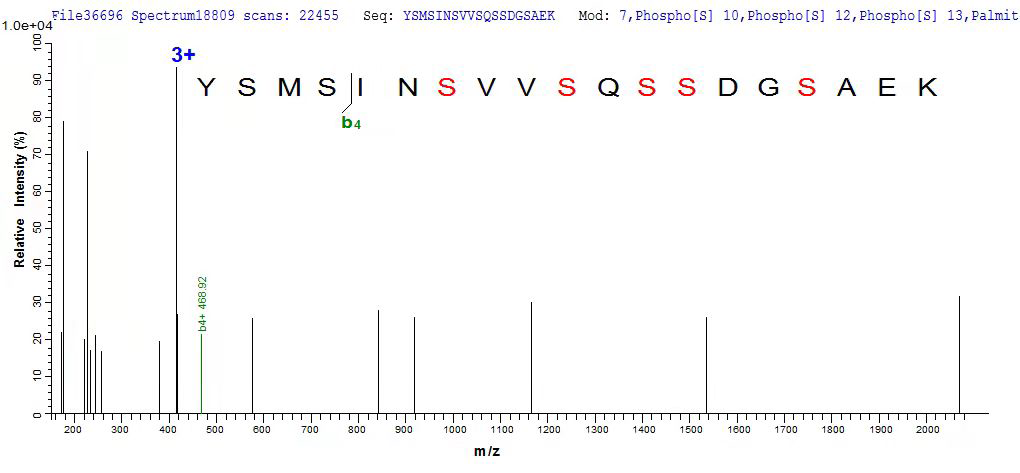

Supplement: S2 Fig — MoRgs7 phosphorylation sites in Guy11 in comparison with the ΔMosep1 mutant expressing MoRgs7 variants. (TIF) [file pgen.1010748.s002.tif]

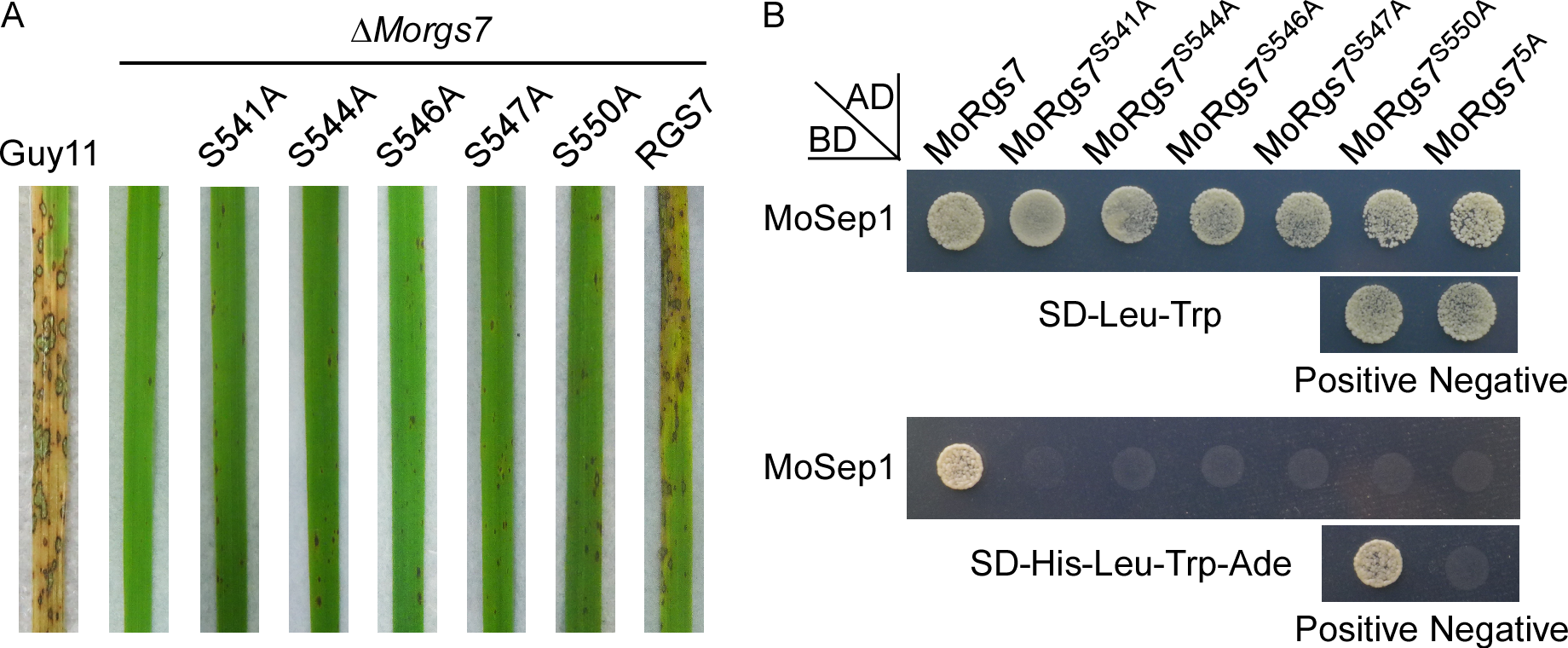

Supplement: S3 Fig — (A) Pathogenicity assay. The experimental procedures were conducted as the same as described for Fig 3. (B) Yeast two-hybrid analysis. MoSep1 was co-introduced with MoRgs7 and its each site-directed mutant into the AH109 strain, respectively. Transformants were plated on SD-Leu-Trp (as control) and SD-Leu-Trp-His-Ade (for further selection) for 5 days. (TIF) [file pgen.1010748.s003.tif]

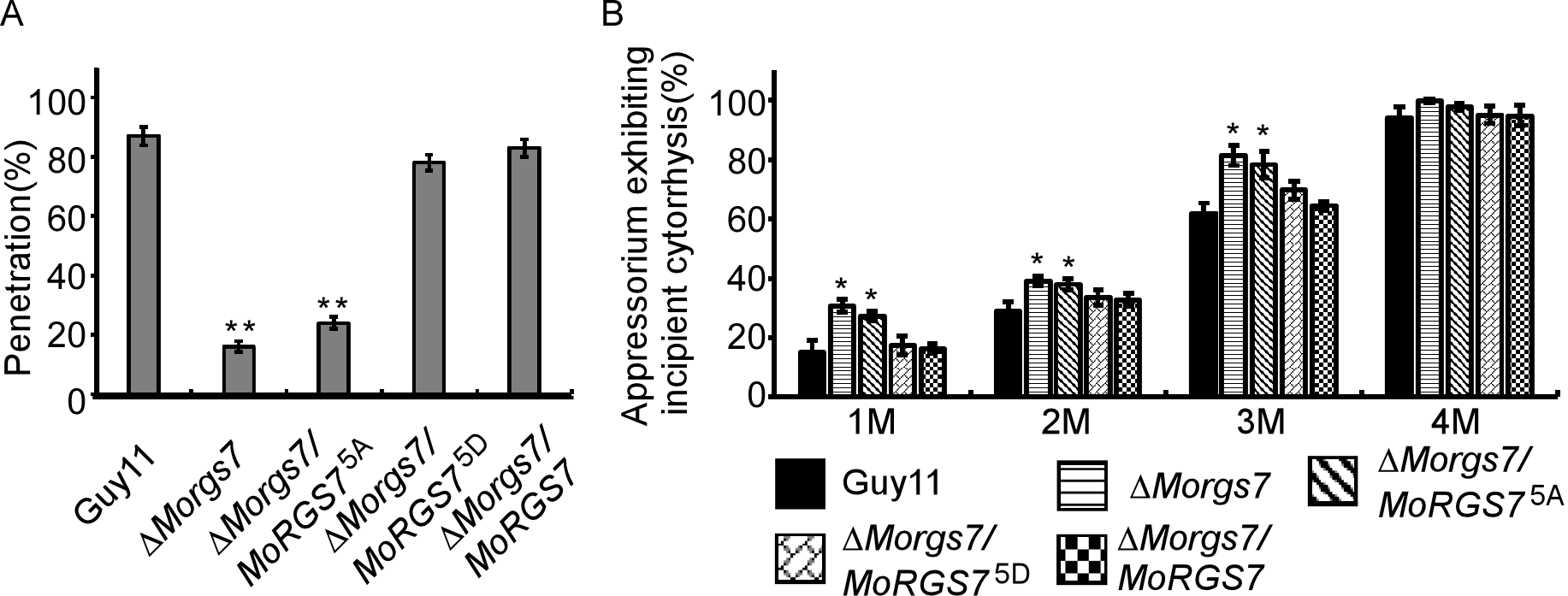

Supplement: S4 Fig — (A) The cytorrhysis assay was conducted by incubating conidia on the hydrophobic surface and treated with various concentrations of glycerol (1–4 M). Numbers of collapsed appressoria among 100 appressoria were recorded and the examination was repeated twice. Error bars represent SDs and asterisks represent significant differences (**p < 0.01). (B) Observation for penetration on the rice sheath. The penetration rate was counted 3 times. Error bars represent SDs and asterisks represent significant differences (*p < 0.05). (TIF) [file pgen.1010748.s004.tif]

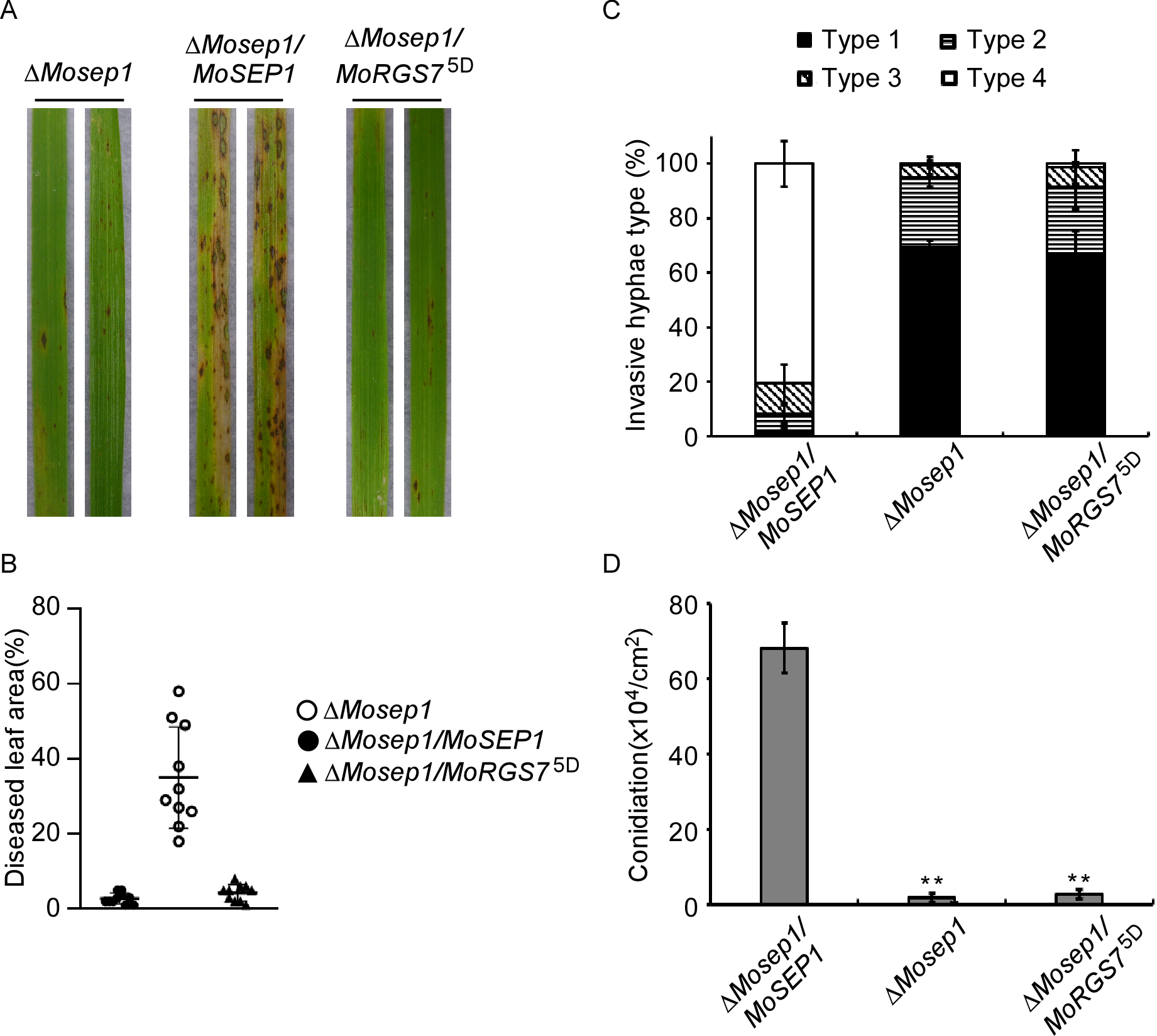

Supplement: S5 Fig — (A, B, and C) Pathogenicity assay, diseased leaf area analysis, and infectious hyphal type assessment were conducted as the same as described for Fig 3. (D) Statistical analysis of conidia. Conidia grown on SDC medium for 7 days in the dark followed by 3 days of continuous fluorescence illumination at 28°C were assessed. Error bars represent SD and asterisks represent significant differences (**p < 0.01). (TIF) [file pgen.1010748.s005.tif]

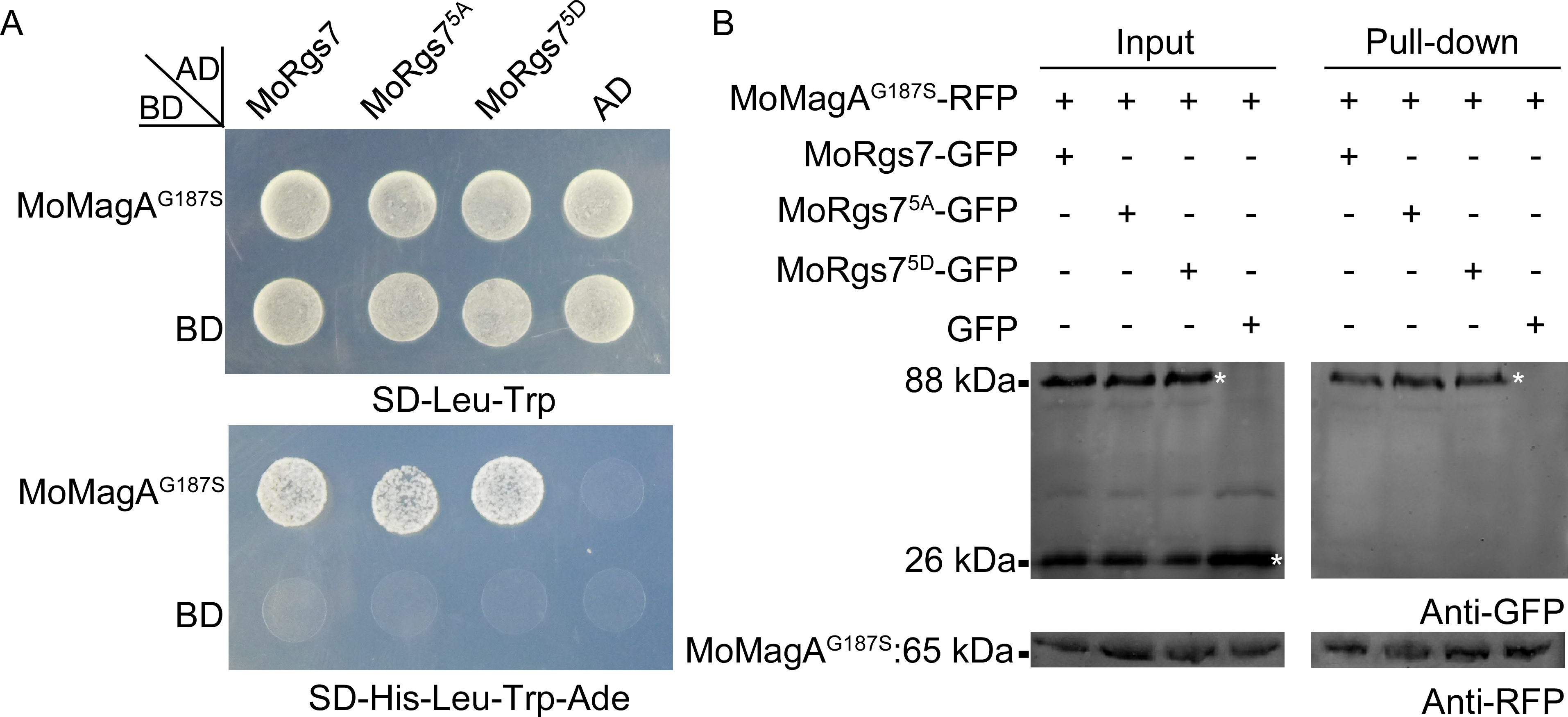

Supplement: S6 Fig — (A) Yeast two-hybrid analysis. MoMagAG187S was co-introduced with MoRgs7 and the site-directed mutagenesis mutants MoRgs75A and MoRgs75D into the AH109 strain. The transformants were plated on SD-Leu-Trp (as control), SD-His-Leu-Trp (for initial selection), and SD-Leu-Trp-His-Ade (for further selection) for 5 days. (B) Co-IP assay for the interaction between MoMagAG187S, MoRgs7 and its site-directed mutagenesis MoRgs75A and MoRgs75D. Co-expression of MoMagAG187S-RFP and MoRgs7-GFP, MoRgs75A-GFP and MoRgs75D-GFP in Guy11, respectively. Proteins were incubated with anti-RFP beads and detected by anti-GFP and anti-RFP antibodies. (TIF) [file pgen.1010748.s006.tif]

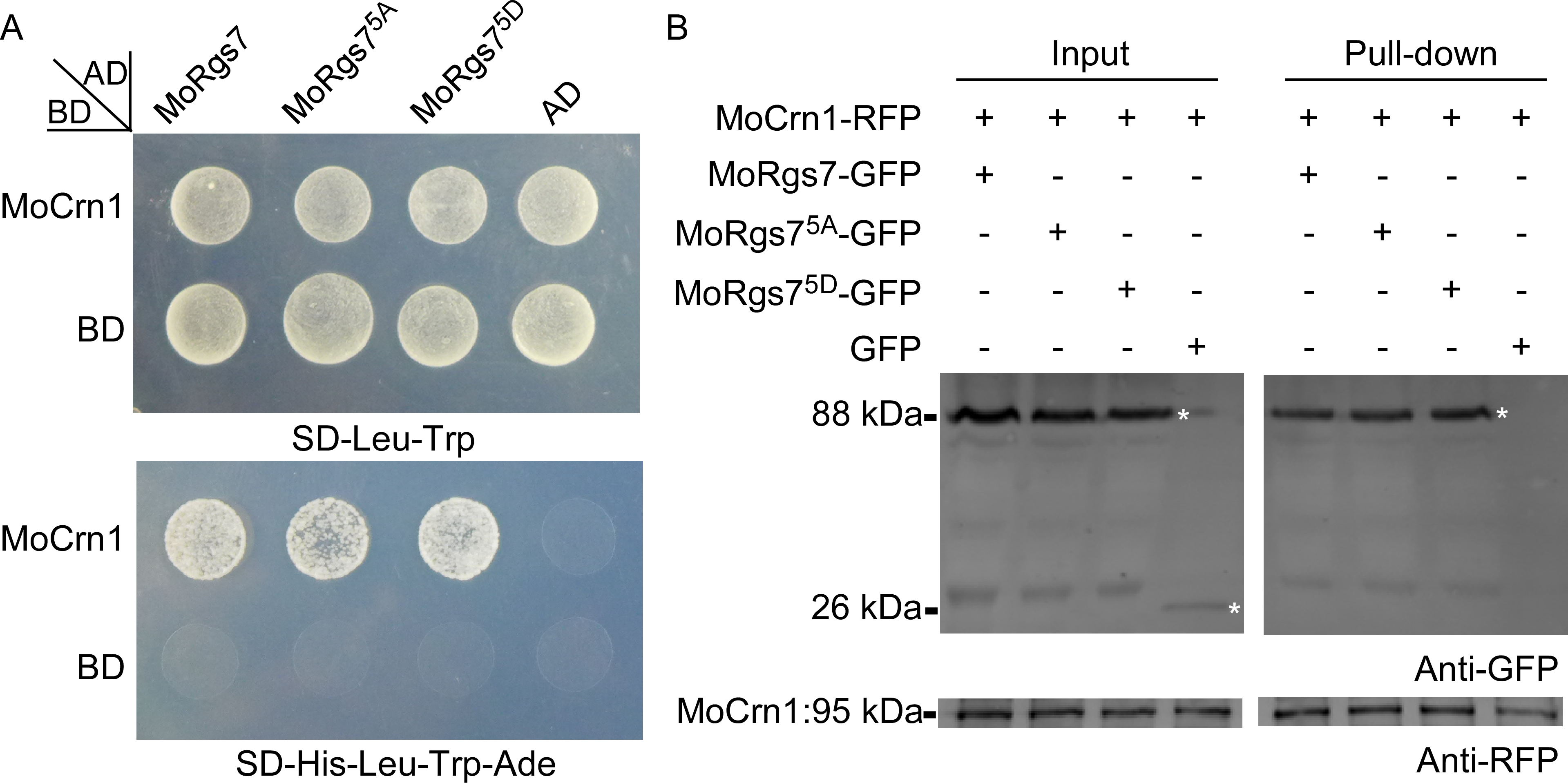

Supplement: S7 Fig — (A) Yeast two-hybrid analysis. MoCrn1 was co-introduced with MoRgs7 and its site-directed mutagenesis MoRgs175A and MoRgs75D variants into AH109 strain, respectively. Transformants were plated on SD-Leu-Trp (as control), SD-His-Leu-Trp (for initial selection), and SD-Leu-Trp-His-Ade (for further selection) for 5 days. (B) Co-IP assay for the interaction between MoCrn1, MoRgs7 and its site-directed mutagenesis MoRgs75A and MoRgs75D. Co-expression of MoCrn1-RFP and MoRgs7-GFP, MoRgs75A-GFP and MoRgs75D-GFP in the wild-type strain Guy11, respectively. Proteins were incubated with anti-RFP beads and detected by anti-GFP and anti-RFP antibodies. (TIF) [file pgen.1010748.s007.tif]

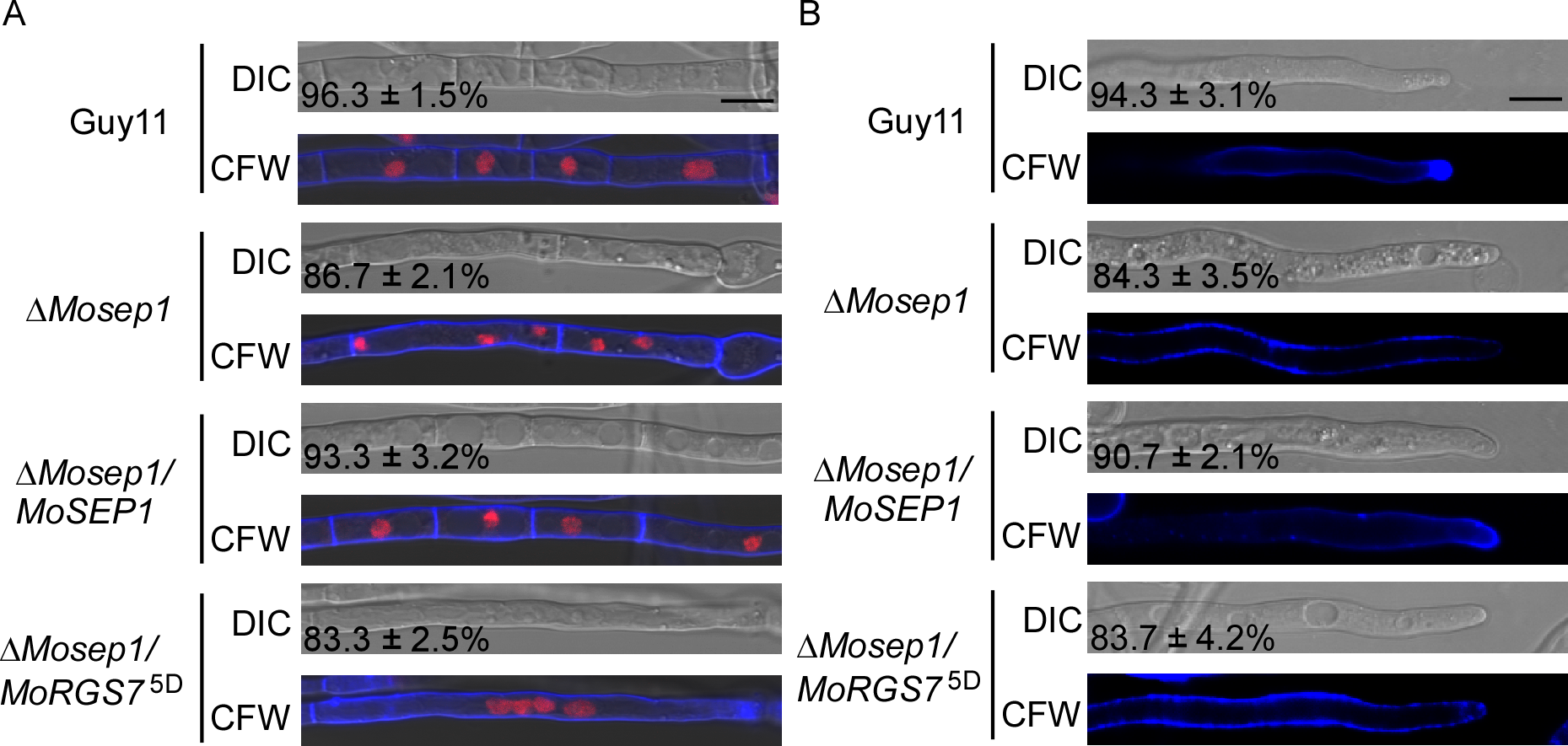

Supplement: S8 Fig — (A) Hyphae of Guy11 and the ΔMosep1, ΔMosep1/MoSEP1, and ΔMosep1/MoRGS75D mutants expressing the H1-RFP construct were stained with CFW and examined by epifluorescence microscopy. Scale bar: 10 μm. (B) Hyphae of Guy11 and the ΔMosep1, ΔMosep1/MoSEP1, and ΔMosep1/MoRGS75D mutants were stained with CFW and examined by epifluorescence microscopy. Percentages shown in all images were calculated by the observation of 100 randomly-selected hyphae, and the observation was repeated twice. Scale bar: 10 μm. (TIF) [file pgen.1010748.s008.tif]
