# Supplementary material for: Hydrophobic cue-induced appressorium formation depends on MoSep1-mediated MoRgs7 phosphorylation and internalization in Magnaporthe oryzae
Source: PLoS Genet. 2023 May 15;19(5):e1010748. doi: 10.1371/journal.pgen.1010748 (PMC10184898; doi:10.1371/journal.pgen.1010748)
Supplement: S1 Text — MoRgs7 was inserted into vector pGBKT7, and was were co-introduced with a yeast two-hybrid cDNA library contained various stages RNS of Magnaporthe oryzae into yeast AH109 strain. Then, AH109 strain was incubated on SD-Leu-Trp and SD-Leu-Trp-His-Ade for 5 days. The names of known protein filled in the brackets. The underlined protein is mentioned in this research. Among this table, MoMagA (MGG_01818) and MoCrn1 (MGG_06389) has been proved been interacted with MoRgs7 in previous studies (Li et al., 2019b; Zhang et al., 2011b). (DOCX) [file pgen.1010748.s009.docx]

S1 Text. Identification of MoRgs7 binding proteins.

| Gene locus | Protein name | Gene locus | Protein name |
| --- | --- | --- | --- |
| MGG_06712 | 5-methyltetrahydropteroyltriglutamate- homocysteine S-methyltransferase | MGG_04719 | guanine nucleotide-binding protein subunit beta-like protein (MoMip11) |
| MGG_01818 | guanine nucleotide-binding protein alpha-3 subunit (MoMagA) | MGG_01084 | glyceraldehyde-3-phosphate dehydrogenase |
| MGG_03185 | ATP synthase subunit beta | MGG_04829 | 60S ribosomal protein L9-A |
| MGG_03641 | elongation factor 1-alpha | MGG_06389 | coronin-6 (MoCrn1) |
| MGG_06958 | Hsp70-like protein | MGG_03982 | actin |
| MGG_15100 | Polyketide synthase | MGG_01722 | adenylyl cyclase-associated protein (MoCap1) |
| MGG_04100 | STE/STE11/CDC15 protein kinase (MoSep1) | MGG_02970 | ubiquitin carboxyl-terminal hydrolase 6 |
| MGG_04503 | carbamoyl-phosphate synthase subunit arginine-specific large | MGG_06044 | ubiquitin-60S ribosomal protein L40 |
| MGG_08063 | Pyruvate kinase | MGG_07752 | ATP synthase subunit alpha |
| MGG_05673 | 40S ribosomal protein S3 | MGG_15774 | Ketol-acid reductoisomerase |
| MGG_01742 | elongation factor 2 | MGG_13065 | SCF E3 ubiquitin ligase complex F-box protein grrA |
| MGG_12805 | tryptophan synthase | MGG_09916 | ATP synthase subunit gamma |
| MGG_14971 | elongation factor 3 | MGG_01756 | ubiquitin-conjugating enzyme E2 2 |
| MGG_07200 | Plasma membrane ATPase | MGG_04118 | fatty acid synthase beta subunit dehydratase |
| MGG_08190 | 60S ribosomal protein L4-A | MGG_05063 | Phosphoglycerate kinase |
| MGG_16149 | ADP, ATP carrier protein | MGG_04978 | E3 ubiquitin ligase complex SCF subunit scon-3 |
